# Second-order regulation: IFN-γ suppresses IL-17A-mediated type 3 inflammation

**DOI:** 10.3389/fimmu.2026.1744476

**Published:** 2026-05-11

**Authors:** Vijay Raaj Ravi, Sophia H. Maxfield, Emma N. Niszczak, Hannah Y. Kim, Olivia S. Harlow, Anjali Kakarla, Kalyn D. Whitehead, Anukul T. Shenoy

**Affiliations:** 1 Dept. of Microbiology and Immunology, University of Michigan Medical School, Ann Arbor, MI, United States; 2 Dept. of Internal Medicine, Division of Pulmonary and Critical Care Medicine, University of Michigan Medical School, Ann Arbor, MI, United States

**Keywords:** CD4^+^ T cells, IFN-γ, IL-17, lung, mucosal immunity, STAT1, T_H_1 cells, T_H_17 cells

## Abstract

**Background:**

T helper 1 (T_H_1) cells often accompany T_H_17 cells across diverse tissues in health and disease, including the lungs. However, roles for the T_H_1 effector cytokine, IFN-γ, in T_H_17-driven type 3 inflammation is unclear.

**Methods:**

We devised a simplified reductionistic model to determine the role of IFN-γ in IL-17A-driven inflammation during *Streptococcus pneumoniae* (*Spn*) infection *in vivo.* Briefly, intratracheal instillation of *Spn* along with recombinant TNF-α and IL-17A was used to mimic rapid *Spn*-specific, T_H_17-driven, type 3 inflammation seen in lungs on memory recall infection with *Spn*. Co-instillation of recombinant IFN-γ was used to probe the role for this T_H_1 cell-derived effector cytokine in anti-*Spn* immune response. Immune cellularity in bronchoalveolar lavage (BAL) was used to determine impacts of IFN-γ on type 3 inflammation in murine airways. Mice sufficient for- or lacking- IFN-γ or STAT1 were used to assess the immunoregulatory functions of IFN-γ *in vivo.*

**Results:**

IFN-γ promptly muted IL-17A-induced inflammatory cell accumulation in *Spn*-infected airways through a STAT1-dependent mechanism. Both female and male mice demonstrated similar anti-inflammatory effects of IFN-γ on type 3 inflammation. We find that the impact of IFN-γ was dependent on the degree of type 3 inflammation such that IFN-γ’s immunoregulatory role became more striking at lower concentrations of all the three cytokines. Of note, the immunoregulatory effect of IFN-γ against T_H_17-driven type 3 inflammation was also evident in physiologically relevant settings: while immunized wild type (WT) mice controlled lethal *Spn* infection, immunized IFN-γ knockout mice exhibited even better *Spn* clearance. This improved antimicrobial resistance, however, was accompanied by heightened airway neutrophilia (which could be phenocopied by mere neutralization of IFN-γ in immunized wild type mice) suggesting risk for immunopathology.

**Conclusions:**

Our findings identify a distinct immunoregulatory mechanism that operates within non-lymphoid tissues, where IFN-γ limits IL-17A-mediated type 3 inflammation via STAT1. Thus, the frequent accompaniment of T_H_17 cells with T_H_1 cells may represent a conserved mechanism that restrains immunopathological potential of T_H_17-driven neutrophilic inflammation via STAT1 signaling in non-lymphoid tissues.

## Introduction

T helper-1 (T_H_1) cells often accompany T_H_17 cells in multiple type 3 inflammatory states across body sites in health and disease (1–4). Some prominent examples include multiple sclerosis in the central nervous system (5), colitis in the colon (6), psoriasis of the skin (7), allergies (8, 9) and microbial infections at barrier epithelial sites (10–16). The reasons for this pairing are incompletely understood. While T_H_1 cell-derived IFN-γ and T_H_17 cell-derived IL-17A inhibit polarization of naïve CD4^+^ T cells to T_H_17 (17) and T_H_1 lineages (18) respectively, several studies have demonstrated that T_H_1 and T_H_17 cells instead synergize to exacerbate disease (2, 19–22). Given these conflicting findings, we sought to resolve the role of IFN-γ using a tractable model of IL-17A-induced type 3 antibacterial inflammation within murine lungs.

*Streptococcus pneumoniae* (*Spn*) is a Gram-positive opportunistic pathogen that frequently colonizes the human nasopharynx and is microaspirated into the lungs of healthy humans globally (23, 24). Despite prolonged spells of colonization and inhalation, most healthy individuals clear this pathobiont asymptomatically (25–29). Induction of T_H_17- and T_H_1-polarized lung tissue resident memory (T_RM_) T cells in response to frequent *Spn* inhalation is thought to be a key driver of this robust antibacterial immunity (16, 30). While *Spn*-specific T_H_17 T_RM_ cells, via IL-17A, coax lung epithelial cells to rapidly recruit neutrophils and clear inhaled *Spn* (16, 30), roles of T_H_1 T_RM_ cells and their effector cytokine IFN-γ within *Spn*-experienced lungs remain unclear. This is a major knowledge gap since *Spn-*specific CD4^+^ T_RM_ cells are capable of serotype-independent protection (16) and lend themselves as promising targets for broadly protective vaccines against all serotypes of *Spn;* the latter being a World Health Organization (WHO) priority pathogen in need of better antibiotics and vaccines due to serotype replacement and antibiotic resistance (24, 31–33).

Here, by intratracheally delivering recombinant IL-17A and IFN-γ (along with *Spn*) to immunologically mimic antigen-dependent reactivation of T_H_17- and T_H_1- T_RM_ cells, we explore whether the T_H_1 effector cytokine IFN-γ may alter IL-17A-driven type 3 inflammation of murine airways. By directly delivering the T cell-effector cytokines to the lung mucosa, we bypass the first-order cross-regulation between T_H_1 and T_H_17 cells that occurs within secondary lymphoid organs (SLOs) (17, 18) to uncover a second-order cross-regulatory mechanism wherein IFN-γ limits IL-17A-mediated type 3 inflammation in a STAT1-dependent manner within the airways. Thus, our findings suggest that T_H_1 biology may have evolved to accompany T_H_17 cells to limit their immunopathological potential at non-lymphoid tissue (NLT) sites.

## Results

### IFN-γ decreases IL-17A-driven neutrophilia in a sex-independent manner

**Figure 1:**
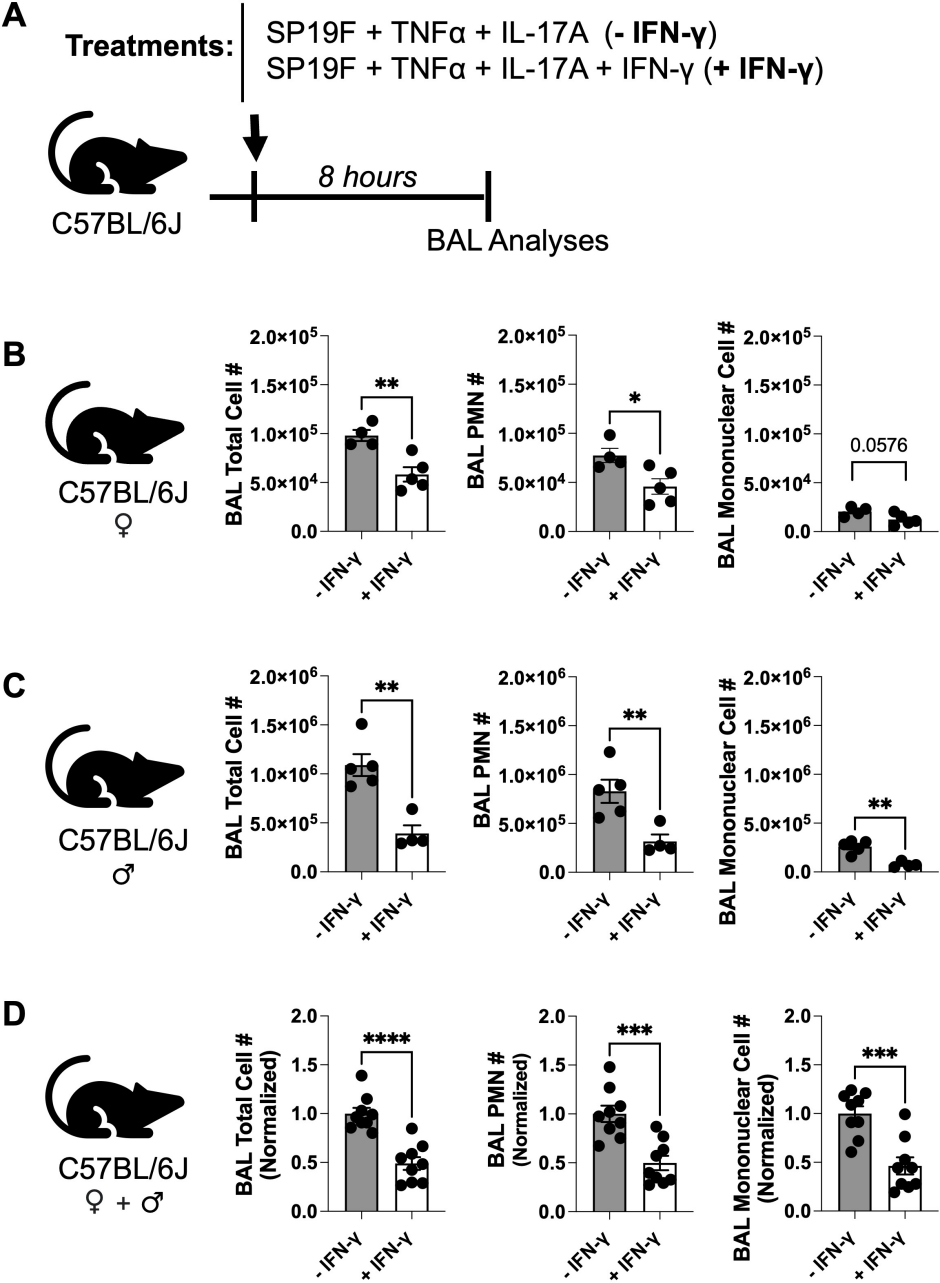
IFN-γ dampens IL-17A-driven airway inflammation in a sex-independent manner. **(A)** Schematic of experimental model used. **(B–D)** Total cell number, PMN number, and mononuclear cell number present in the bronchoalveolar lavages (BAL) 8 hours post treatment in **(B)** Female wild type (WT) mice, **(C)** Male WT mice, and **(D)** Aggregated female and male WT mice data normalized to (-IFN-γ) treatment group within each sex. Unpaired t test. *p* value: *≤ 0.05, **≤ 0.01, ***≤ 0.001, ****≤ 0.0001. All data have n≥4 mice, 2 experiments, mean ± SEM. Cell numbers were enumerated using differential staining of cytospins made from BALs.

Recurrent inhalation of *Spn* induces a profound expansion of tissue-resident T_H_17- and T_H_1-polarized T_RM_ cells amongst all IL-17A and IFN-γ expressing lymphocytes within experienced lungs (**Supplementary Figures S1A–C**) (16, 30). While these T_H_17 T_RM_ cells rapidly recruit neutrophils to drive robust antimicrobial immunity (14, 16, 34), roles of T_H_1 T_RM_ cells within *Spn*-experienced lungs remain unclear. We sought to investigate whether the T_H_1 effector cytokine IFN-γ plays any roles in modulating IL-17A-induced type 3 inflammation within such *Spn*-experienced murine lungs. Informed by knowledge that T_H_1 and T_H_17 T_RM_ cells coexist in *Spn*-experienced lungs in comparable numbers (**Supplementary Figures S1A–C**) (16, 30) and secrete similar levels of IFN-γ or IL-17A in response to *Spn* reencounter, respectively (16, 30), we developed a simplified reductionist *in vivo* model to mimic rapid anti-*Spn*-memory recall. Briefly, naïve C57BL/6J mice were intratracheally administered a cocktail of 200ng of recombinant TNF-α and IL-17A (along with a non-lethal isolate of *Spn* belonging to serotype 19F, Sp19F) to mimic rapid T_H_17 T_RM_ response against homeostatic inhalation of *Spn* (**Figure 1A**). Immune cellularity of the airways was assessed in bronchoalveolar lavage (BAL) 8 hours post-infection when T_H_17 T_RM_ cell-driven airway neutrophilia is robust within *Spn*-experienced murine lungs (16, 34). Of note, instilled cytokine cocktails included- or lacked- 200ng of recombinant IFN-γ to model presence or absence of *Spn*-specific T_H_1 T_RM_ cells respectively. We included *Spn* in all our cytokine cocktails to reproduce a scenario of an *Spn*-reinfection that would activate antigen-specific T_H_17 and T_H_1 T_RM_ cells to profusely secrete their effector cytokines TNF-α, IL-17A and IFN-γ in *Spn*-experienced lungs during memory recall (16, 30). Hereafter, the treatment group that received IFN-γ will be referred to as “+ IFN-γ” whereas the group without IFN-γ will be called “- IFN-γ”. By delivering the T_H_ effector cell cytokines directly at the lung mucosa, this model bypasses the first-order cross-regulation between T_H_1 and T_H_17 cells that occurs within SLOs (17, 18) and lends a powerful system to probe for roles of IFN-γ in altering IL-17A-driven type 3 inflammation in the lung mucosa where comparable numbers of T_H_1 and T_H_17 T_RM_ cells reside post their egress from SLOs (16, 30). Consistently, both female (**Figure 1B**) and male mice (**Figure 1C**) co-administered IFN-γ showed reduced accumulation of total, polymorphonuclear (PMNs), and mononuclear cells compared to their “- IFN-γ” counterparts (**Figure 1D**, compiled and normalized). Thus, our data suggest that the T_H_1 cytokine IFN-γ reduces IL-17A-driven influx of PMNs and mononuclear cells in *Spn*-infected mouse airways, independent of sex.

### IFN-γ exerts stronger immunomodulatory effects at lower cytokine doses

**Figure 2:**
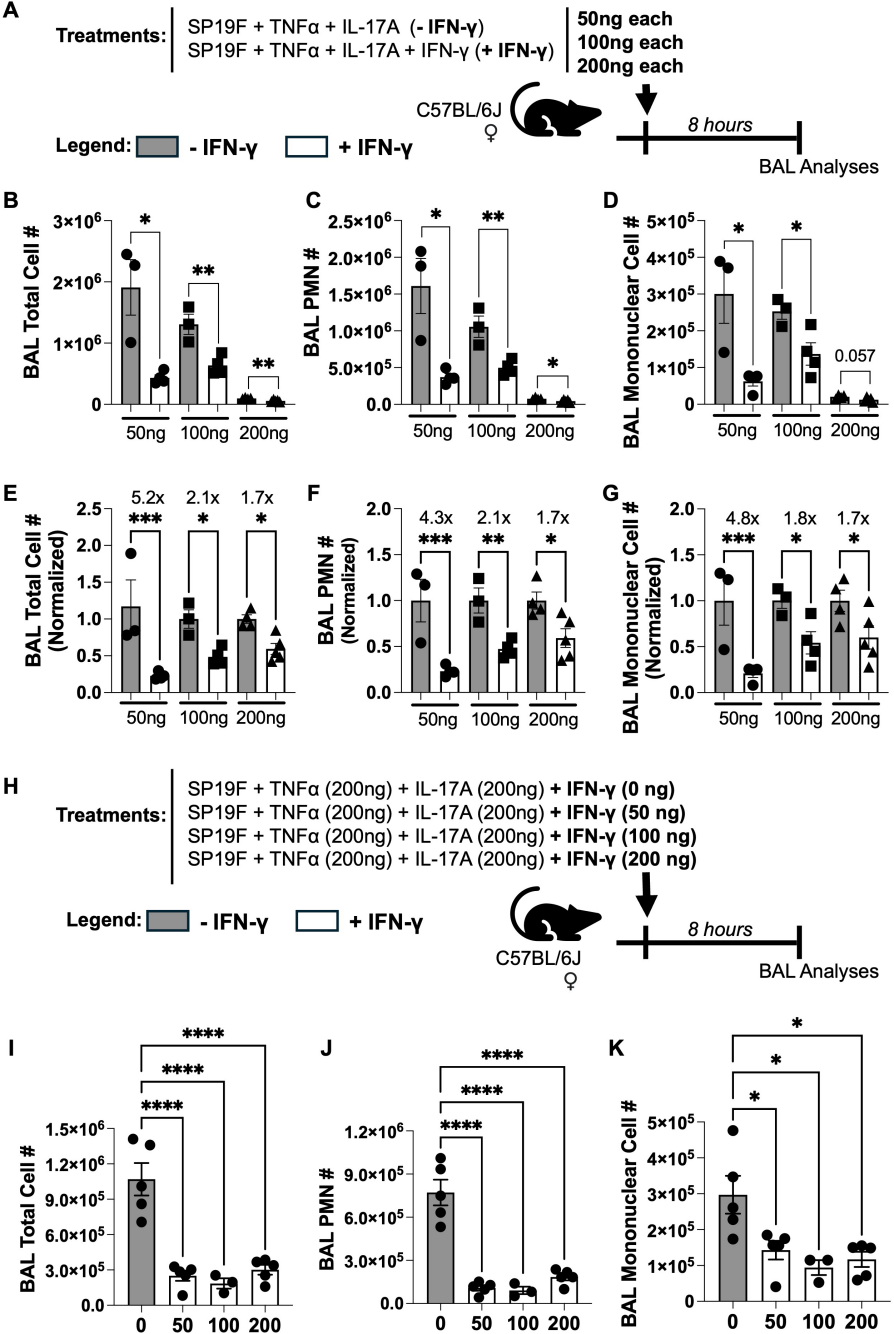
Lower doses of IFN-γ exhibit greater immunoregulatory effects. **(A)** Schematic of experimental model used. **(B)** Total cell number, **(C)** PMN cell number, and **(D)** Mononuclear cell number in BALs from female WT mice 8 hours post treatment with varying cytokine concentrations. **(E)** Total cell number, **(F)** PMN cell number, and **(G)** Mononuclear cell number in BALs from female WT mice 8 hours post treatment with varying specified concentrations normalized to (-IFN-γ) treatment group. Unpaired t test. **(H)** Schematic of experimental model used. **(I)** Total cell number, **(J)** PMN cell number, and **(K)** Mononuclear cell number in BALs from female WT mice 8 hours post treatment with specific cocktails. Ordinary one-way ANOVA with Fisher’s LSD test. *p* value: *≤ 0.05, **≤ 0.01, ***≤ 0.001, ****≤ 0.0001. All data have n≥3 mice, 2 experiments, mean ± SEM. Cell numbers were enumerated using differential staining of cytospins made from BALs.

Multiple cytokines function in a concentration-dependent manner where higher levels of the cytokines exert stronger biological effects (35, 36). Given that the 200ng doses we used are much higher than the physiologically relevant levels of these cytokines that accumulate in *Spn*-experienced lungs in response to memory recall (16), we asked if reducing the levels of these cytokines would extinguish the immunoregulatory effect of IFN-γ. We intratracheally administered cocktails of 50ng, 100ng, or 200ng of each cytokine along with Sp19F followed by analyses for BAL cellularity (**Figure 2A**). Surprisingly, the immunoregulatory effects of IFN-γ were markedly enhanced at lower concentrations of the cytokines (**Figures 2B–G**). While 50ng cocktails showed a 5.2-fold decrease in BAL total cellularity driven by IFN-γ, 200ng cocktails instead exhibited only a 1.7-fold decrease (**Figures 2B, E**). A similar dose-dependent effect was observed for both PMN (**Figures 2C, F**) and mononuclear cell accumulation in the airways (**Figures 2D, G**). Next, to assess if different doses of IFN-γ may suppress IL-17A-driven airway inflammation to different degrees, we intratracheally administered all mice with constant dose of TNF-α and IL-17A (200ng each) but instilled varying doses of IFN-γ (0ng, 50ng, 100ng, or 200ng) along with Sp19F followed by analyses for BAL cellularity (**Figure 2H**). All doses of IFN-γ reduced BAL total cellularity (**Figure 2I**), PMNs (**Figure 2J**), and mononuclear cell accumulation (**Figure 2K**) equally well. Thus, our data suggest that IFN-γ can suppress IL-17A-driven airway inflammation even at low concentrations and that IFN-γ’s immunomodulatory effect may be magnified at lower concentrations of all the cytokines. Given the stark suppression seen at 50ng doses of all cytokines, we hereafter use 50ng cocktails for the rest of our study unless otherwise specified. Of note, the immunoregulatory effect of IFN-γ was independent of *Spn* instillation since IFN-γ dampened IL-17A-driven enhanced PMN infiltration into the airways even when administered in absence of bacteria (**Supplementary Figures S2A, B**). What is more, IFN-γ mediated this immunoregulatory effect via mechanisms distinct from IFN-γ- or IL-17A- secreting innate lymphocytes (**Supplementary Figures S3A, B**) or IL-17A-driven canonical PMN chemoattracts (**Supplementary Figures S3C–G**) as evidenced by comparable levels of these cells and chemokines irrespective of IFN-γ instillation. Interestingly, IFN-γ increased CXCL2 accumulation in the airways when delivered as part of the 200ng cocktail (**Supplementary Figure S3G**), further supporting the idea that IFN-γ restrains IL-17A-driven neutrophilia by mechanisms other than suppressing IL-17A-target neutrophil chemoattractant production.

### IFN-γ broadly mutes type 3 inflammatory cell landscape

**Figure 3:**
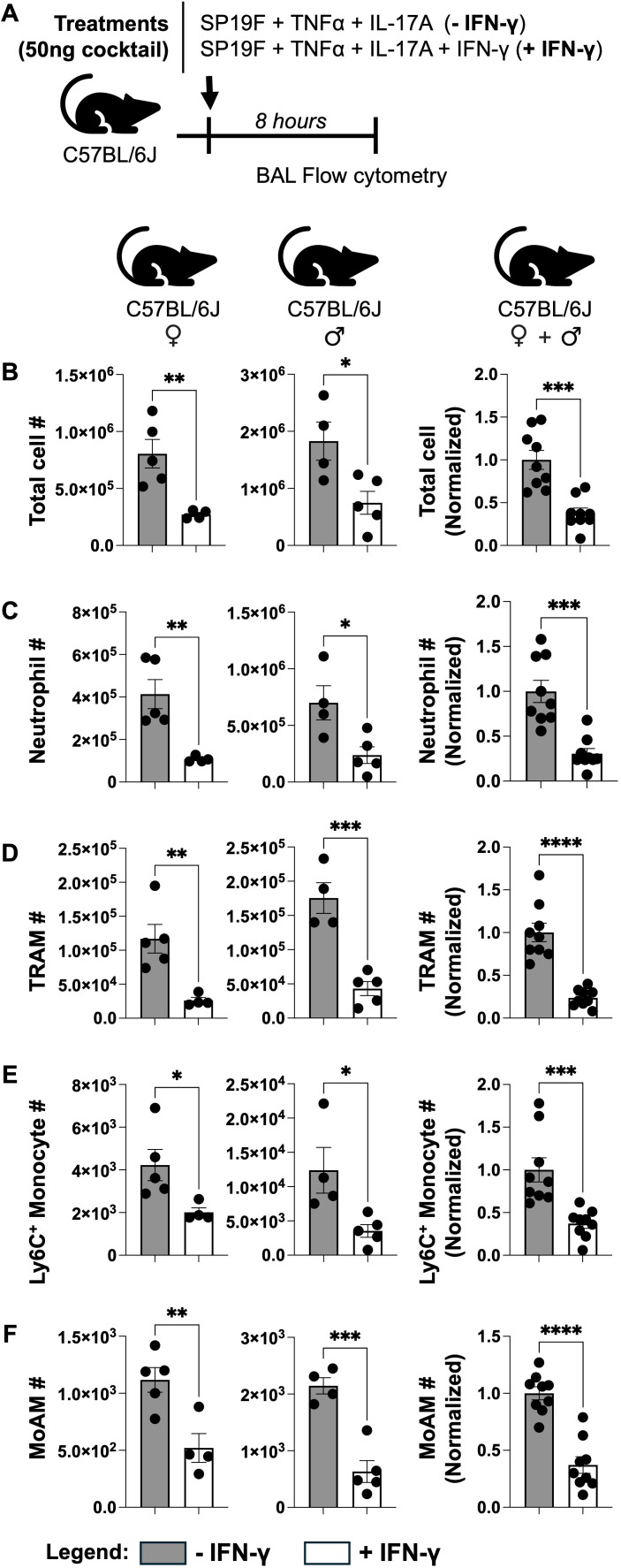
IFN-γ broadly dampens the type 3 inflammatory cell landscape. **(A)** Schematic of experimental model used. **(B)** Total cellularity, **(C)** Neutrophils, **(D)** TRAMs, **(E)** Ly6C+ Monocytes, and **(F)** MoAMs in BALs of female and male WT mice 8 hours post treatment as enumerated using flow cytometry. Fold changes in aggregated data were calculated relative to the -IFN-γ treatment group within each sex. Unpaired t test. *p* value: *≤ 0.05, **≤ 0.01, ***≤ 0.001, ****≤ 0.0001. All data have n≥3 mice, 2 experiments, mean ± SEM.

IL-17A-driven neutrophilic inflammation is accompanied by enhanced accrual of Ly6C^+^ monocytes (37) and macrophages in the inflamed tissue (38). To assess the extent of immunoregulatory effect of IFN-γ on IL-17A-driven inflammatory cell landscape, we performed flow cytometry on BAL (**Figure 3A**, gating strategy in **Supplementary Figure S4**). In addition to reducing total cellularity (**Figure 3B**) and neutrophils (**Figure 3C**), IFN-γ consistently reduced the numbers of multiple type 3 inflammatory cells including tissue resident alveolar macrophages (TRAMs) (**Figure 3D**), Ly6C^+^ monocytes (**Figure 3E**), and monocyte-derived alveolar macrophages (MoAMs) (**Figure 3F**) that accumulated within inflamed airways of mice independent of sex. Beyond the aforementioned cell types, we also expanded our analyses to include other key innate immune cell subsets that may be impacted by IFN-γ. While IFN-γ impacted accumulation of Ly6C^-^ monocytes (**Supplementary Figures S5F, M**), and CD11b^-^Ly6C^+^ DCs (**Supplementary Figures S5B, I**) in sex-dependent fashion, it consistently decreased numbers of CD11b^+^Ly6C^+^ DCs (**Supplementary Figures S5C, J**) across both sexes of mice. Numbers of conventional type 1 DCs (cDC1s), CD11b^+^Ly6C^-^ dendritic cells (CD11b^+^ DCs), eosinophils, and lymphocytes remained unaffected (**Supplementary Figure S5**). Thus, our data suggest that IFN-γ remodels the inflammatory cell landscape of murine airways experiencing type 3 inflammation. To determine the degree to which IFN-γ may remodel this IL-17A-driven type 3 inflammation, we repeated the experiment but included a group of mice that received only TNF-α and IFN-γ without IL-17A (**Supplementary Figure S6A**). Consistent with the notion that IFN-γ potently restrains IL-17A-mediated type-3 inflammation, we found that numbers of neutrophils (**Supplementary Figure S6B**), Ly6C+ monocytes (**Supplementary Figure S6D**) and MoAMs (**Supplementary Figure S6E**) were comparable in airways of mice receiving IFN-γ, whether they received IL-17A or not. Taken together, our data strengthen the conclusion that IFN-γ potently inhibits IL-17A-driven type 3 inflammatory cell infiltration in the murine airways.

### IFN-γ signaling via STAT1 inhibits type 3 inflammation

**Figure 4:**
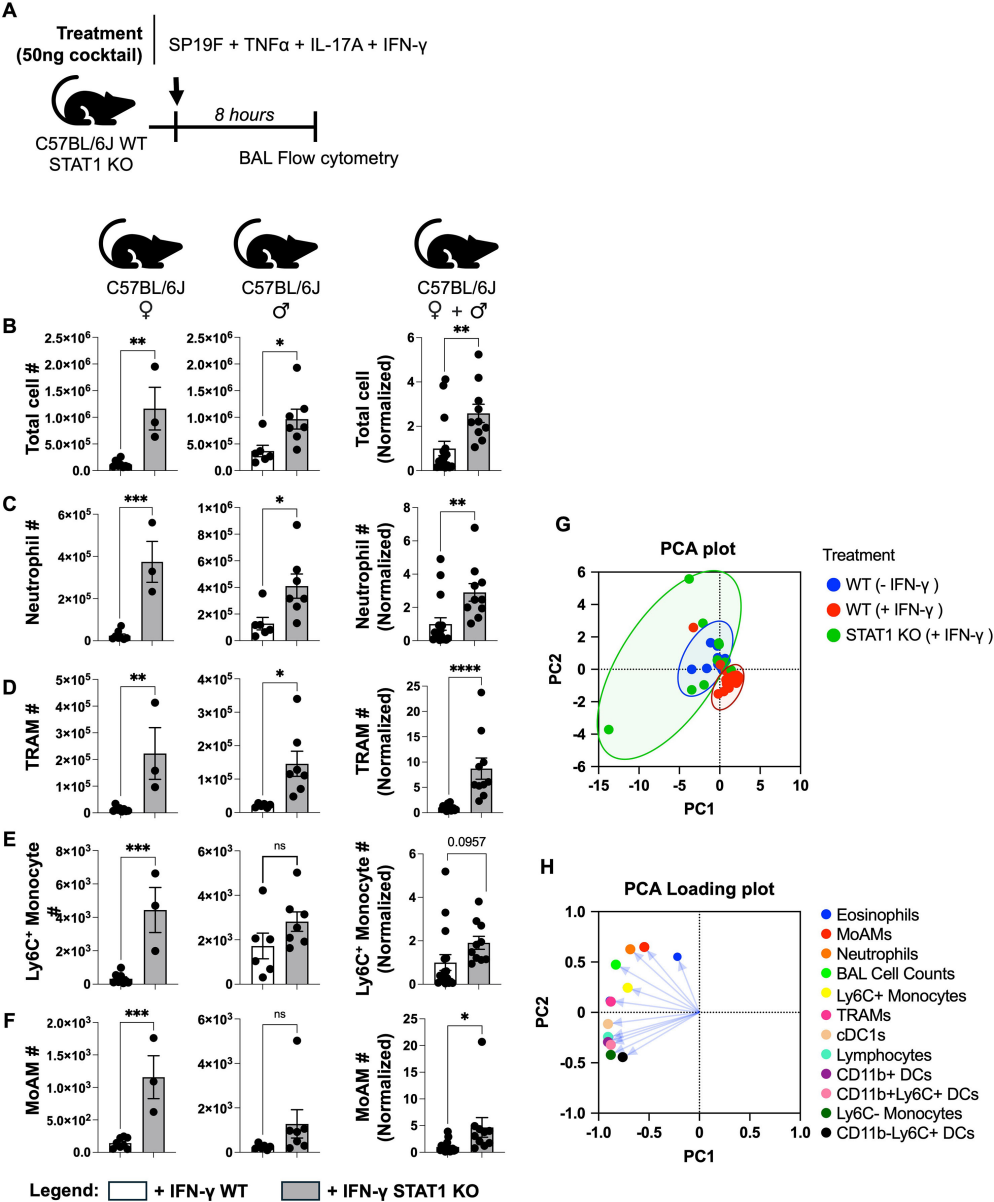
IFN-γ suppresses type 3 inflammation through STAT1. **(A)** Schematic of experimental model used. **(B)** Total cell number, **(C)** Neutrophil, **(D)** TRAMs **(E)** Ly6C+ Monocytes, and **(F)** MoAMs in BALs of female and male WT and STAT1 KO mice 8 hours post treatment as enumerated using flow cytometry. Fold changes in aggregated data were calculated relative to the -IFN-γ treatment group within each sex. Unpaired t test. **(G)** Principal component analysis (PCA) plot of compiled BAL cellularity data from C57BL/6J WT and STAT1 KO mice based on total cell numbers and 11 distinct cell types identified by flow cytometry. **(H)** PCA loadings indicating the magnitude and direction of each variable’s contribution to a principal component. *p* value: *≤ 0.05, **≤ 0.01, ***≤ 0.001, ****≤ 0.0001. All data have n≥3 mice, 2 experiments, mean ± SEM.

IFN-γ conventionally signals via the Janus kinase (JAK)–signal transducer and activator of transcription 1 (STAT1) pathway to exert its effects (39). We sought to determine whether STAT1 is required for IFN-γ’s immunoregulatory activity within lung mucosa during type 3 inflammation (**Figure 4A**). To determine this, we intratracheally instilled cocktails of all three cytokines at 50 ng each (along with Sp19F) into wild type (WT) and STAT1 knockout mice and measured airway cellularity using flow cytometry on BAL. Consistently, all the key inflammatory cell types associated with type 3 airway inflammation were elevated in airways of both female and male STAT1 KO mice despite IFN-γ instillation (**Figures 4B–F**). In addition to these, numbers of cDC1s (**Supplementary Figures S7A, H**) were also elevated in mice lacking STAT1 independent of their sex. Thus, our data suggest that STAT1 deficiency may phenocopy the type 3 inflammatory landscape displayed by WT mice lacking IFN-γ instillation. Indeed, *post-hoc* concatenation of data from WT mice receiving 50ng of TNF-α and IL-17A (i.e. - IFN-γ WT) with data from WT and STAT1 KO mice receiving 50ng of all three cytokines (i.e. +IFN-γ WT and +IFN-γ STAT1 KO), followed by Principal Component Analysis (PCA) to reduce the dimensionality of our data revealed that STAT1 KO mice clustered close to the “- IFN-γ WT” group and away from “+IFN-γ WT” group (**Figure 4G**). We next queried the cell types that influenced the direction and magnitude of each principal component. Among all the cell types identified, PCA loading plot revealed that the major type 3 inflammatory cells (namely neutrophils, TRAMs, Ly6C^+^ monocytes, and MoAMs) drove the co-clustering towards the top left quadrant (**Figure 4H**) reflecting strong positive correlations and indicating their primary contribution in driving separation of STAT1 KO mice towards “- IFN-γ WT” and away from “+IFN-γ WT” mice in the PCA. Other contributors to the clustering appeared to be eosinophils and cDC1s, suggesting their dependence on IFN-γ-STAT1 signaling during type 3 inflammation (**Figure 4H**; **Supplementary Figures S5**, **7**). Collectively, these findings demonstrate that IFN-γ mediates its immunoregulatory effects on lung mucosa during type 3 inflammation in a STAT1-dependent manner.

### IFN-γ impairs bacterial clearance while restricting neutrophilic inflammation

**Figure 5:**
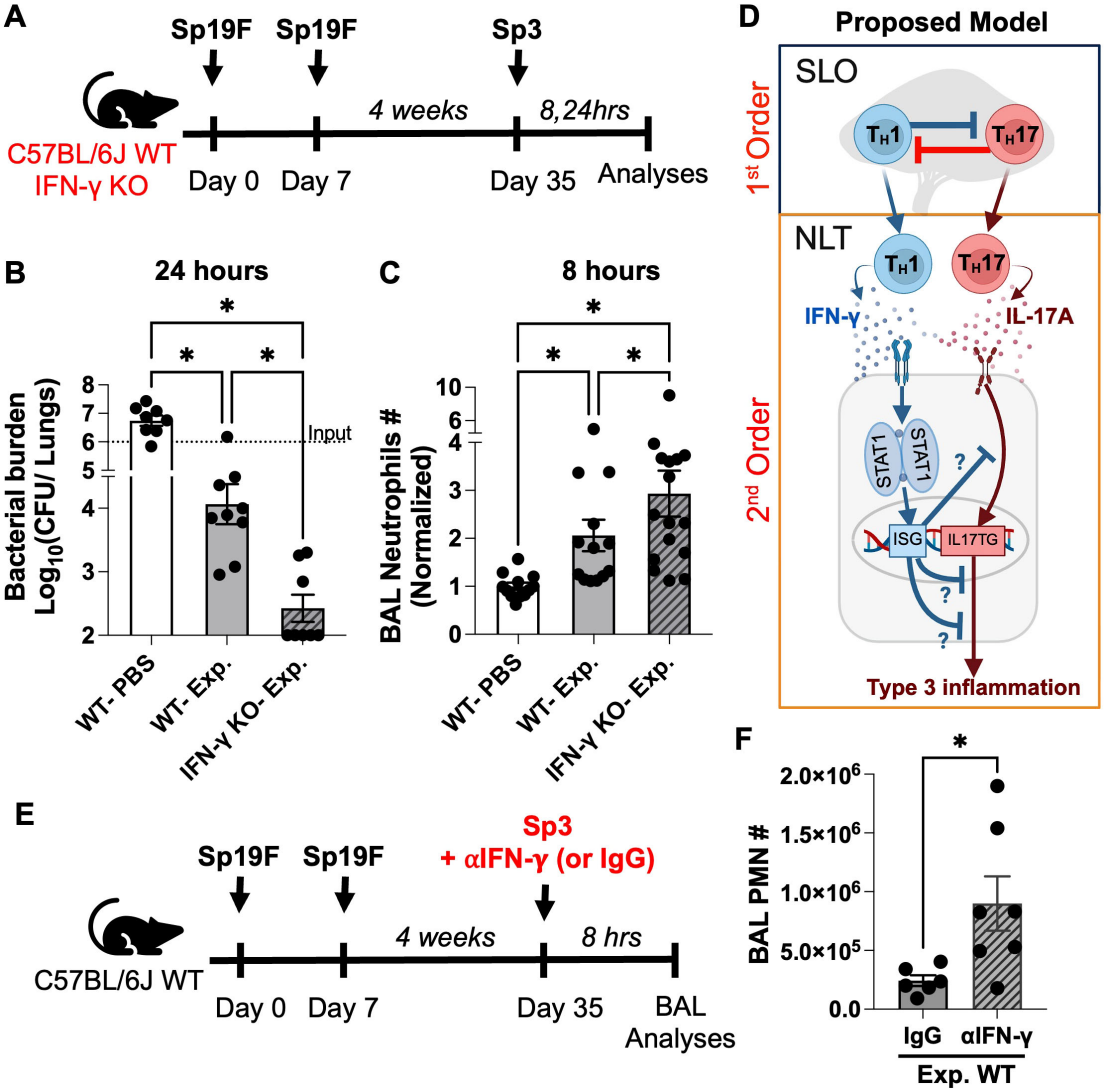
IFN-γ limits neutrophilic inflammation and compromises bacterial clearance on memory recall. **(A)** Schematic of experimental model used. **(B)** *Spn* serotype 3 CFU counts 24 hours post infection (hpi). Dotted line indicates the bacterial load in the challenge inoculum. Ordinary one-way ANOVA with two-stage step-up method of Benjamini, Krieger, and Yekutieli to correct for multiple comparisons. FDR q value: *≤0.05. **(C)** Neutrophil numbers in BAL of naive and *Spn*-experienced WT and IFN-γ KO mice 8 hpi as enumerated using flow cytometry. Lognormal ordinary one-way ANOVA with two-stage step-up method of Benjamini, Krieger, and Yekutieli to correct for multiple comparisons. FDR q value: *≤0.05. Cell numbers are normalized to their numbers observed within naïve WT mice in the same experiment. **(D)** Graphical abstract illustrating our working model of second-order cross-regulation between type 1 and type 3 inflammatory cytokines within non-lymphoid tissues (NLTs). ISG: Interferon stimulated genes, IL17TG: IL-17 target genes. The question marks indicate potential mechanisms by which IFN-γ may inhibit IL-17A driven type 3 inflammation (see discussion for details). **(E)** Schematic of experimental model used. **(F)** Total PMN number present in the bronchoalveolar lavages (BAL) 8 hours post treatment as enumerated using differential staining of cytospins. All data have n≥3 mice, 2–3 experiments, mean ± SEM.

Lung-resident T_H_17 T_RM_ cells use IL-17A to coax local epithelial cells (34) and fibroblasts (40) to rapidly recruit neutrophils within 7–8 hours of memory recall to confer robust antimicrobial immunity in the lungs (16, 34). We asked if T_H_1 T_RM_ cell-derived IFN-γ may dampen this T_H_17-driven protective inflammation. We intratracheally administered Sp19F into WT and IFN-γ-deficient (IFN-γ KO) mice on days 0 and 7 to generate T_RM_ cells within the lungs (16, 34); control WT mice received sterile PBS. Following 4 weeks of rest, all groups were intratracheally challenged with a lethal serotype mismatched strain of *Spn* belonging to serotype 3 (Sp3) and bacterial clearance was assessed (**Figure 5A**). Strikingly, while experienced WT mice cleared Sp3 by 100-fold within 24 hours of infection, the experienced IFN-γ KO mice cleared the bacteria by ~5000-fold within 24 hours (**Figure 5B**). This enhanced microbial clearance was accompanied by greater neutrophil accumulation within airways of *Spn-*experienced IFN-γ KO mice compared to *Spn-*experienced IFN-γ sufficient WT mice (**Figure 5C**). No such increments were observed for other immune cells (**Supplementary Figures S8A–J**) or IL-17A-target neutrophil chemokines (**Supplementary Figures S8K–N**) that may explain this enhanced neutrophilic influx and bacterial clearance. Our data, thus, suggest that IFN-γ secreted by T_H_1 T_RM_ cells may restrain T_H_17 T_RM_ cell-driven neutrophilic infiltration and compromise robust antimicrobial immunity via mechanisms that are distinct from suppressing the IL-17A-target neutrophil chemokines. However, this interpretation ignores an important caveat that loss of IFN-γ releases constrains on T_H_17 polarization within SLO (17)(depicted in top half of **Figure 5D**) which presented as increased numbers of circulating T_H_17 cells in blood of *Spn-*experienced IFN-γ KO mice (**Supplementary Figures S9A, B**). Thus, although this proclivity was not as prominent for T_H_17 T_RM_ cells in the *Spn-*experienced IFN-γ KO mouse lungs (**Supplementary Figure S9C**), it is plausible that the expanded T_H_17 pools in *Spn-*experienced IFN-γ KO mice contributed to higher IL-17A levels in the lungs (**Supplementary Figure S9D**), explaining the enhanced airway neutrophilia seen in **Figure 5C**. Thus, to explicitly test if T_H_1 T_RM_ cell-derived IFN-γ restrains T_H_17 T_RM_ cell driven neutrophilic inflammation on memory recall without impacting numbers of T_H_1 and T_H_17 T_RM_ cells, we directly neutralized IFN-γ in experienced WT mice using neutralizing antibody concomitant to Sp3 challenge. Consistent with our other data, WT mice receiving the IFN-γ neutralizing antibody exhibited muted neutrophil recruitment to the lungs despite *Spn*-experience (**Figures 5E, F**). Thus, our data suggest that T_H_1 T_RM_ cell-derived IFN-γ restrains T_H_17 T_RM_ cell-driven neutrophilic inflammation to dampen antibacterial immunity in experienced lungs.

## Discussion

Herein, we directly delivered recombinant IFN-γ and IL-17A cytokines at the lung mucosa to find that the T_H_1 effector cytokine IFN-γ limits IL-17A-mediated type 3 inflammation in a STAT1-dependent- and sex-independent-manner; these regulatory effects of IFN-γ were more pronounced at lower concentrations of all the cytokines. We find that IFN-γ potently inhibited accumulation of various myeloid cells involved in type 3 inflammation including tissue resident alveolar macrophages (TRAMs), neutrophils, Ly6C^+^ monocytes, and monocyte-derived AMs (MoAMs). Finally, using *Spn*-experienced IFN-γ-deficient mice and *Spn*-experienced WT mice whose IFN-γ was neutralized, we demonstrate that IFN-γ restricts T_H_17 T_RM_ cell-driven airway neutrophilia with negative consequences on antibacterial immunity within experienced lungs.

T_H_1 cells consistently accompany T_H_17 cells across multiple body sites during various states of health and disease (1–4) ranging from infections (10–16, 41–43), autoimmunity (1, 44, 45), allergy (8, 9, 46, 47), and fibrosis (48, 49) in both mice and humans. However, the significance of this pairing beyond SLOs where they inhibit polarization of naïve T cells into the opposite lineage (17, 18) has remained unclear. Our data suggest that T_H_1 cells, via their key effector cytokine IFN-γ, regulate T_H_17-driven type 3 inflammation within the non-lymphoid tissues. These findings partially explain why mice deficient in type 1 immunity consistently have worse outcomes in myriad models of type 3 inflammatory disease including experimental autoimmune encephalitis (EAE) model of MS (50), tuberculosis (51, 52), bacterial pneumonia (53) and allergies (8, 54). T_H_17 cells incite their inflammatory activities by stimulating local epithelial cells (34) and fibroblasts (40) to produce neutrophil attracting chemokines via IL-17 signaling. Given that mere instillation of recombinant IFN-γ into naive lungs was sufficient to rapidly mute IL-17A-driven inflammation without impacting numbers of existing IL-17A or IFN-γ secreting innate lung lymphocytes, our data suggest that IFN-γ may exert its immunoregulatory effect via an extra-lymphocytic mechanism that involves signaling into tissue resident cells within non-lymphoid tissues. We propose that pairing of T_H_17 cells with T_H_1 cells represents an evolutionarily conserved strategy to ensure just enough type 3 inflammation to control the antigenic insult while simultaneously imposing restraints on the immunopathological potential of T_H_17 cells. The price that our immune system pays for this measured type 3 inflammation is more modest antimicrobial clearance which can only reach its full potential when IFN-γ-mediated constraints are lifted. Supporting this, previous studies with *Mycobacterium tuberculosis* show that mice lacking IFN-γ receptor experienced T_H_17-driven excessive neutrophilic infiltration and lethal immunopathology within infected lungs (52); mere depletion of neutrophils in these mice was sufficient to improve survival suggesting that the overt immunopathology, and not uncontrolled bacteria, was the cause of death. Thus, efforts towards developing T_H_17 T_RM_ cell-directed vaccines should consider the yin-yang relationship between type 1 and type 3 cytokines and should consider promoting T_H_1 T_RM_ cells concomitantly to balance antimicrobial immunity and risk for immunopathology. Taken together, our findings fundamentally extend our knowledge regarding cross-regulation between type 1 and type 3 inflammatory cells beyond the SLOs to identify a distinct, second-order regulatory mechanism that operates within non-lymphoid tissues where tissue resident cells integrate type 1 and type 3 effector cytokine signals to fine-tune inflammation (**Figure 5D**). We anticipate this tissue resident cell to be a non-myeloid cell since a recent study found that myeloid cell STAT1 was dispensable for regulation of type 3 neutrophilic inflammation in a *Klebsiella pneumoniae* driven bacterial pneumonia (53). We predict this could include at least three mutually non-exclusive (if not all three) potential mechanisms: (1) IFN-γ-induced STAT1 product/s may somehow directly dampen IL-17A signaling, (2) IFN-γ-induced STAT1 product/s may somehow impact stability of IL-17A targets, or (3) IFN-γ-induced STAT1 product/s may somehow prevent the recruitment of inflammatory cells downstream of IL-17A signaling by mechanism distinct from alterations in IL-17A-target neutrophil attracting chemokines. Precisely how IFN-γ signaling mutes IL-17A-driven inflammation and in which tissue resident cell/s is an active area of investigation with significant translational and clinical potential.

Our results find that IFN-γ signaling suppresses type 3 inflammation via its conventional transcriptional factor STAT1. The significance of STAT1 in suppressing IL-17-driven inflammation is evident from clinical observations that individuals with STAT1 gain-of-function (GOF) mutations are unable to control mucosal infections by common pathobionts such as *Candida albicans* (55, 56)*, S. pneumoniae* (57, 58)*, Staphylococcus aureus* (57, 58)*, Pseudomonas aeruginosa* (57, 58)*, and Haemophilus influenzae* (57, 58); all agents that require T_H_17 derived IL-17 and resulting type 3 inflammation for timely control. While the role for lymphocyte-intrinsic STAT1 in precluding protective T_H_17 cell development within SLOs of these patients is well recognized (17), our findings suggest an additional extra-lymphocytic layer of dysregulation where overt STAT1 activation within tissue resident cells of such patients may mute neutrophil recruitment to render type 3 inflammation insufficient for microbial control at mucosal barriers. Indeed, >25% of patients with STAT1 GOF mutations who receive hematopoietic stem cell transplantation (HSCT, to normalize immune-cell intrinsic STAT1) die from infections within few years post-HSCT (58). Instead, broad inhibition of IFN signaling with JAK inhibitors improve symptoms and lead to complete recovery in such patients (58, 59). It is tempting to posit that JAK inhibitors outperform HSCT owing to their ability to agnostically inhibit IFN signaling within hematopoietic and non-hematopoietic cells; the latter would be unaltered by HSCTs.

Immunomodulatory effects of IFN-γ are most pronounced at lower cytokine concentrations. Previous studies have found that lower concentrations of IFN-γ compromises the induction of optimal antimicrobial activity of neutrophils (35), as well as natural killer cells and T cells (36). Our data instead find that IFN-γ suppresses IL-17A-driven inflammation equally well at low concentrations. This stands in stark contrast to IFN-γ’s immunostimulatory activity that requires high concentrations of the cytokine (35, 36). What is more, we also find that IFN-γ’s immunoregulatory effect is weakened at higher concentrations of TNF-α and IL-17A; a condition where we found surprising reductions in numbers of airway infiltrating cells overall (when compared with lower doses of TNF-α and IL-17A). While the exact mechanisms underlying this observation are unclear, our findings suggest that IFN-γ’s immunoregulatory activity may be at its most potent at lower concentrations of TNF-α and IL-17A that are closer to physiological levels produced during healthy immunity. This also implies that tissues suffering from severe type 3 inflammation and secreting overt levels of TNF-α and IL-17A (such as those seen in severe or chronic diseases) may be less responsive to IFN-γ mediated suppression of T_H_17-driven type 3 inflammation. Instead, in such contexts, IFN-γ may synergize with IL-17A to worsen immunopathology by compromising the epithelial barrier integrity (60, 61) and overtly activating innate and adaptive immune cells as observed in diseases such as inflammatory bowel disease (19, 62), autoimmune thyroiditis (63, 64), diabetes (65), among others. Thus, in addition to highlighting the importance of hormesis as a key determinant of cytokine biology, our findings encourage caution with use of- and interpretation of data from- experiments that employ high levels of cytokines to study downstream biology; these systems may reflect chronic pathological conditions. Direct comparative studies assessing effects of IFN-γ at different concentrations of all the cytokines in our model are now warranted.

Beyond IFN-γ, our studies have implications for type 1 (IFN-I) and type 3 IFN (IFN-III) biology as well, owing to their dependence on STAT1 (66, 67). While their STAT1-dependent inhibitory effects at low-grade inflammation may explain IFN-I and IFN-III’s suppressive effects of neutrophilic inflammation in some contexts (68–70), their weakened immunoregulatory activity at high-grade inflammation may explain their starkly contrasting and detrimental effects during severe/chronic infections (71–73) and autoimmunity (74). It is tempting to posit that patients with autoantibodies against IFN-I (and thus insufficient STAT-1 signaling) experience worsened SARS-CoV-2 driven airway neutrophilia leading to overt lung immunopathology and severe disease (75, 76).

Taken together, using a simplified reductionist model of lung-resident *Spn*-specific T_RM_ cell memory response that bypasses first-order T_H_1 and T_H_17 cell cross-regulation in SLOs (17, 18), we uncover a second-order, cross-regulatory mechanism where IFN-γ limits IL-17A-mediated type 3 inflammation via STAT1 within non-lymphoid tissues. While our findings, when viewed in context of T cell immunology, suggest that T_H_1 cells may have evolved to accompany T_H_17 cells to limit their immunopathological potential at peripheral tissue sites, they also reveal a more broadly applicable and fundamental phenomenon of second-order cross-regulation that implicates immunoregulatory roles for IFN-γ secreting innate lymphocytes in restraining type 3 inflammation provoked by other IL-17A-producing cells. This expanded view of cytokine cross-regulation highlights the importance of context, site, and dose in immune-modulation, and opens new avenues for next generation therapeutic strategies that will finely balance protective and pathological inflammation at barrier tissue sites.

## Material and methods

### Lead contact and materials availability

Correspondence and requests for information, resources and reagents should be directed to Anukul T. Shenoy (anukuls@umich.edu). All data will be made available by the corresponding authors upon reasonable request.

### Mice

6-week-old C57BL/6J (stock# 000664), IFN-γ KO (B6.129S7-Ifng^tm1Ts^/J, Stock# 002287), and STAT1 KO (B6.129S(Cg)-Stat1^tm1Dlv^/J, Stock# 012606) mice were obtained from Jackson labs (USA) and subsequently bred in-house to ensure acclimation to local conditions. Mice aged 7–14 weeks were used for all experiments. Animals were housed in a specific pathogen-free facility on a 12-hour light/dark cycle with ad libitum access to standard chow and water. Euthanasia was performed using isoflurane overdose, and death was confirmed via pneumothorax prior to organ collection. All animal procedures conformed to the guidelines approved by the Institutional Animal Care and Use Committee at the University of Michigan, Ann Arbor.

### Intratracheal cytokine and *Streptococcus pneumoniae* administration

Mice were administered 200ng, 100ng, or 50ng of recombinant mouse TNF-α (315-01A-20UG, Peprotech), IL-17A (Cat# 210-17-25UG, Peprotech), and/or IFN-γ (Cat# 315-05-100UG, Peprotech) along with ~10^6^ CFU of serotype 19F *Spn* (Sp19F, Strain EF3030) in 100μL PBS for intratracheal instillation models mimicking antigen-experienced mice.

### Mice CFU experiments

*Spn* experienced mice were generated as previously described (16, 34). Mice were anesthetized via isoflurane and were intratracheally infected with either 1-5 × 10^6^ CFU of serotype 19F *Spn* (Strain EF3030) suspended in 100μL of sterile PBS or just 100μL of sterile PBS (control/naïve group) on days 0 and 7 and then allowed to recover for 35 days. On day 36, the mice were intratracheally challenged with 1 × 10^6^ CFU of serotype 3 *Spn* (Sp3, ATCC 6303), suspended in 100μL of sterile PBS. 24 hours after the challenge the mice were euthanized using isoflurane overdose and the lungs were used to enumerate CFUs.

### IFN-γ neutralization

IFN-γ was neutralized using 100μg of anti-mouse IFN-γ antibody (Clone XMG1.2, BioXCell, Cat #BE0055) or isotype IgG1 antibody (Clone HRPN, BioXCell, Cat # BE0088) delivered to mice intraperitoneally 30 minutes prior to Sp3 challenge and intratracheally simultaneously with the Sp3 challenge.

### Bronchoalveolar lavage collection and analyses

Euthanized mice were exsanguinated, and their tracheas cannulated with an 18-gauge cannula followed by 8 rounds of lavage with sterile PBS. The cell pellets from BALs were used to perform cytospins or flow cytometry, and airway cellularity enumeration.

### Bronchoalveolar lavage cytology

Cell pellets were deposited on Fisherbrand™ Superfrost™ Plus Microscope Slides (Cat# 12-550-15, ThermoFisher Scientific) for cytospins using a cytocentrifuge. Slides were air-dried overnight before staining with Hema 3™ Stat Pack solutions. The staining protocol involved dipping the slides for 6 seconds in fixative, 12 seconds in solution I, and 12 seconds in solution II, followed by rinsing with deionized water. Slides were then air-dried for an additional day prior to imaging with an Olympus BX60 microscope.

### Lung digestion for flow cytometry

To enumerate of extravascular versus intravascular lymphocytes, anesthetized mice were retro-orbitally administered 2μg anti-CD45.2 antibody 3–5 min prior to euthanasia (77). Lungs were collected in RPMI-1640 with 10% FBS for flow cytometry. Single-cell suspensions were prepared by digestion of lungs in type 2 collagenase as previously described (78). Lung single-cell suspensions were stimulated *ex vivo* in 24-well plate with 100ng/mL Phorbol Myristate Acetate (PMA)(Sigma Aldrich) and 1μg/mL Ionomycin (Sigma, St. Louis, MO) in T cell stimulation media with Monensin (Biolegend, Cat# 420701) and Brefeldin A (Biolegend, Cat# 420601) both at 1X final concentration for 4 hours at 37 °C and 5% CO_2_. Where applicable, cells were processed for intracellular cytokine staining (ICS) using eBioscience Intracellular Fixation & Permeabilization Buffer Set (Cat# 88-8824-00) as per manufacturer’s protocols.

### Flow cytometry

Flow cytometric analysis on BAL cells and lung cells from mice was performed using the Cytek Aurora or the BD LSRFortessa™ Cell Analyzer. The antibodies used to stain the samples are APC anti-mouse/human CD11b (Clone M1/70, Biolegend, Cat# 101212); PE-Cy7 anti-mouse CD11c (Clone N418, Biolegend, Cat# 117318); Alexa Flour 700 anti-mouse CD45 (Clone 30-F11, Biolegend, Cat# 103128; PE anti-mouse Ly-6G (Clone 1A8, Biolegend, Cat# 127608; Ly-6C Monoclonal Antibody (Clone HK1.4, Invitrogen, Cat# 48-5932-82; APC-Cy™7 anti-Mouse Siglec-F (Clone E50 – 2440, BD Pharmingen, Cat# 565527, 7-AAD (BD Pharmingen, Cat# 559925), PerCP-Cy5.5 anti-mouse CD45 (Clone 30-F11, Biolegend, Cat# 103132), BV510 anti-mouse CD45.2 (Clone 104, Biolegend, Cat# 109838), PE-Cy7 anti-mouse CD3e (Clone 145-2C11, Biolegend, Cat# 100320), Alexa Fluor 488 anti-mouse TCR-γ/δ (Clone GL3, Biolegend, Cat #118127), BUV805 anti-mouse CD8 (Clone 53-6.7, BD Biosciences, Cat# 612898), BV605 anti-mouse CD4 (Clone GK1.5, Biolegend, Cat# 100451), APC anti-mouse IL-17A (Clone TC11-18H10.1, Biolegend, Cat# 506916), APC-Cy7 anti-mouse IFN-*γ* (Clone XMG1.2, Biolegend, Cat# 505850) and Zombie violet (Biolegend, Cat# 423114). Data was analyzed with FlowJo software (BD Biosciences). Gating strategies are detailed in the **Supplementary Figures** and were established using Fluorescence-Minus-One (FMO) controls.

### ELISA

BAL CXCL1, CXCL2, CXCL5 and CCL20, and lung IL-17A levels were measured using ELISA kits from R&D Systems using the manufacturer’s protocols.

### Statistical analyses

Statistical analyses were conducted using Prism 10 (version 10.3.0, GraphPad). Statistical significance was defined as a p value depicted in GraphPad style. Details regarding sample size, number of experimental replicates, and statistical tests applied are provided in each figure legend. Data are presented as mean ± SEM for all figures, except for supplementary **Supplementary Figures 1B** and **4**, which display flow cytometry gating strategy.

## Data Availability

The raw data supporting the conclusions of this article will be made available by the authors, without undue reservation.

## Supplementary material

Supplementary Figure S1 *Spn*-experience seeds murine lungs with tissue-resident T_H_17 and T_H_1 T_RM_ cells. **(A)** Schematic of experimental model used. To accurately discriminate lung resident lymphocytes that are extravascular from circulating lymphoyctes that are intravascular, anesthetized mice administered 2μg anti-CD45.2 antibody 3–5 min prior to lung collection. **(B)** Flow cytometry gating strategy for distinguishing various cytokine-secreting lung resident lymphocyte populations as identified using intracellular cytokine staining (ICS). **(C)** Absolute numbers of ICS-positive tissue-resident (ivCD45-) lymphocytes in naïve versus *Spn*-experienced lungs. Unpaired t test. *p* value: *≤ 0.05. All data have n≥3 mice, 2 experiment, mean ± SEM.

Supplementary Figure S2 IFN-γ inhibits IL-17A-driven enhanced PMN infiltration into the airways even in absence of *Spn*. **(A)** Schematic of experimental model used. **(B)** Total PMN numbers present in BAL of mice 8 hpi treatment with specified cytokine cocktail without Sp19F. Ordinary one-way ANOVA with Fisher’s LSD test. *p* value: *≤ 0.05, **≤ 0.01, ***≤ 0.001, ****≤ 0.0001. All data have n≥3 mice, 2 experiments, mean ± SEM. Cell numbers were enumerated using differential staining of cytospins made from BALs.

Supplementary Figure S3 Instillation of IFN-γ does not impact numbers of innate lymphocytes or IL-17A-driven canonical PMN chemoattracts. **(A)** Schematic of experimental model used. **(B)** Absolute numbers of ICS-positive tissue-resident (ivCD45-) lymphocytes in lungs exposed to distinct cocktails. Unpaired t test. **(C)** Schematic of experimental model used. Levels of **(D)** CXCL1, **(E)** CXCL2, **(F)** CXCL5, and **(E)** CCL20 in BALs from female WT mice 8 hours post treatment with varying cytokine concentrations. Unpaired t test. *p* value: *≤ 0.05. All data have n≥3 mice, 2 experiments, mean ± SEM.

Supplementary Figure S4 Flow cytometry gating strategy for distinguishing various myeloid cells populations.

Supplementary Figure S5 Extended myeloid cell analyses in the BAL of WT mice 8 hours post treatment with cytokine cocktails. Numbers of **(A)** type 1 conventional dendritic cells (cDC1), **(B)** CD11b^-^Ly6C^+^ DCs, **(C)** CD11b^+^Ly6C^+^ DCs, **(D)** CD11b+ DCs, **(E)** eosinophils, **(F)** Ly6C- monocytes, **(G)** lymphocytes in female mice with or without IFN-γ treatment. Unpaired t test. Numbers of **H)** cDC1, **(I)** CD11b^-^Ly6C^+^ DCs, **(J)** CD11b^+^Ly6C^+^ DCs, **(K)** CD11b+ DCs, **(L)** eosinophil, **(M)** Ly6C- monocyte, **(N)** lymphocyte in male mice with or without IFN-γ treatment. Unpaired t test. *p* value: *≤ 0.05, **≤ 0.01, ***≤ 0.001, ****≤ 0.0001. All data have n≥3 mice, 2 experiments, mean ± SEM.

Supplementary Figure S6 IFN-γ potently restrains IL-17A-mediated type-3 inflammation. **(A)** Schematic of experimental model used. Total **(B)** neutrophils, **(C)** TRAMs, **(D)** Ly6C+ Monocytes, and **(E)** MoAMs in BALs of WT mice 8 hours post treatment as enumerated using flow cytometry. Ordinary one-way ANOVA with Fisher’s LSD test. *p* value: *≤ 0.05, **≤ 0.01, ***≤ 0.001, ****≤ 0.0001. All data have n≥3 mice, 2 experiments, mean ± SEM.

Supplementary Figure S7 Extended myeloid cell analyses in the BAL of WT and STAT1 KO mice 8 hours post treatment with cytokine cocktails. Numbers of **(A)** type 1 conventional dendritic cells (cDC1), **(B)** CD11b^-^Ly6C^+^ DCs, **(C)** CD11b^+^Ly6C^+^ DCs, **(D)** CD11b+ DCs, **(E)** eosinophils, **(F)** Ly6C- monocytes, **(G)** lymphocytes in both WT and STAT1 KO female mice with IFN-γ treatment. Unpaired t test. Numbers of **(H)** cDC1, **(I)** CD11b^-^Ly6C^+^ DCs, **(J)** CD11b^+^Ly6C^+^ DCs, **(K)** CD11b+ DCs, **(L)** eosinophils, **(M)** Ly6C- monocytes, **(N)** lymphocytes in both WT and STAT1 KO male mice with IFN-γ treatment. Unpaired t test. *p* value: *≤ 0.05, **≤ 0.01, ***≤ 0.001, ****≤ 0.0001. All data have n≥3 mice, 2 experiment, mean ± SEM.

Supplementary Figure S8 *Spn-*experienced IFN-γ KO and WT mice show comparable numbers of myeloid cells and neutrophil attracting chemokines. **(A)** TRAM, **(B)** Ly6C+ Monocyte, **(C)** MoAM, **(D)** type 1 conventional dendritic cells (cDC1), **(E)** CD11b^-^Ly6C^+^ DC, **(F)** CD11b^+^Ly6C^+^ DC, **(G)** CD11b+ DC, **(H)** Ly6C- monocyte, **(I)** eosinophil, and **(J)** lymphocyte numbers in BAL of naive and *Spn*-experienced WT and IFN-γ KO mice 8 hpi as enumerated using flow cytometry. Lognormal ordinary one-way ANOVA with two-stage step-up method of Benjamini, Krieger, and Yekutieli to correct for multiple comparisons. FDR q value: *≤0.05. Cell numbers are normalized to their numbers observed within naïve WT mice in the same experiment. Levels of **(K)** CXCL1, **(L)** CXCL2, **(M)** CXCL5, and **(N)** CCL20 in of naive and *Spn*-experienced WT and IFN-γ KO mice 8 hpi. Ordinary one-way ANOVA with Fisher’s LSD test, *p* value: *≤ 0.05. All data have n≥3 mice across 2–3 experiments, mean ± SEM.

Supplementary Figure S9 *Spn-*experienced IFN-γ KO mice inherently exhibit enhanced T_H_17 biology. **(A)** Schematic of experimental model used. **(B, C)** Frequency and absolute numbers of IL-17A positive **(B)** circulating (ivCD45+) T_H_17 cells, and **(C)** tissue-resident (ivCD45-) T_H_17 cells in the naive and *Spn*-experienced WT and IFN-γ KO at homeostasis. **(D)** Levels of IL-17A proteins in lungs of naive and *Spn*-experienced WT and IFN-γ KO mice 24 hours post infection with Sp3. Cytokine data is normalized to IL-17A observed within naïve WT mice in the same experiment. Unpaired t test. *p* value: *≤ 0.05, **≤ 0.01, ***≤ 0.001, ****≤ 0.0001. All data have n≥3 mice across 2–3 experiments, mean ± SEM.
