# Supplementary figures and images for: Second-order regulation: IFN-γ suppresses IL-17A-mediated type 3 inflammation
Source: Front Immunol. 2026 May 11;17:1744476. doi: 10.3389/fimmu.2026.1744476 (PMC13199338; doi:10.3389/fimmu.2026.1744476)

## Slide 1
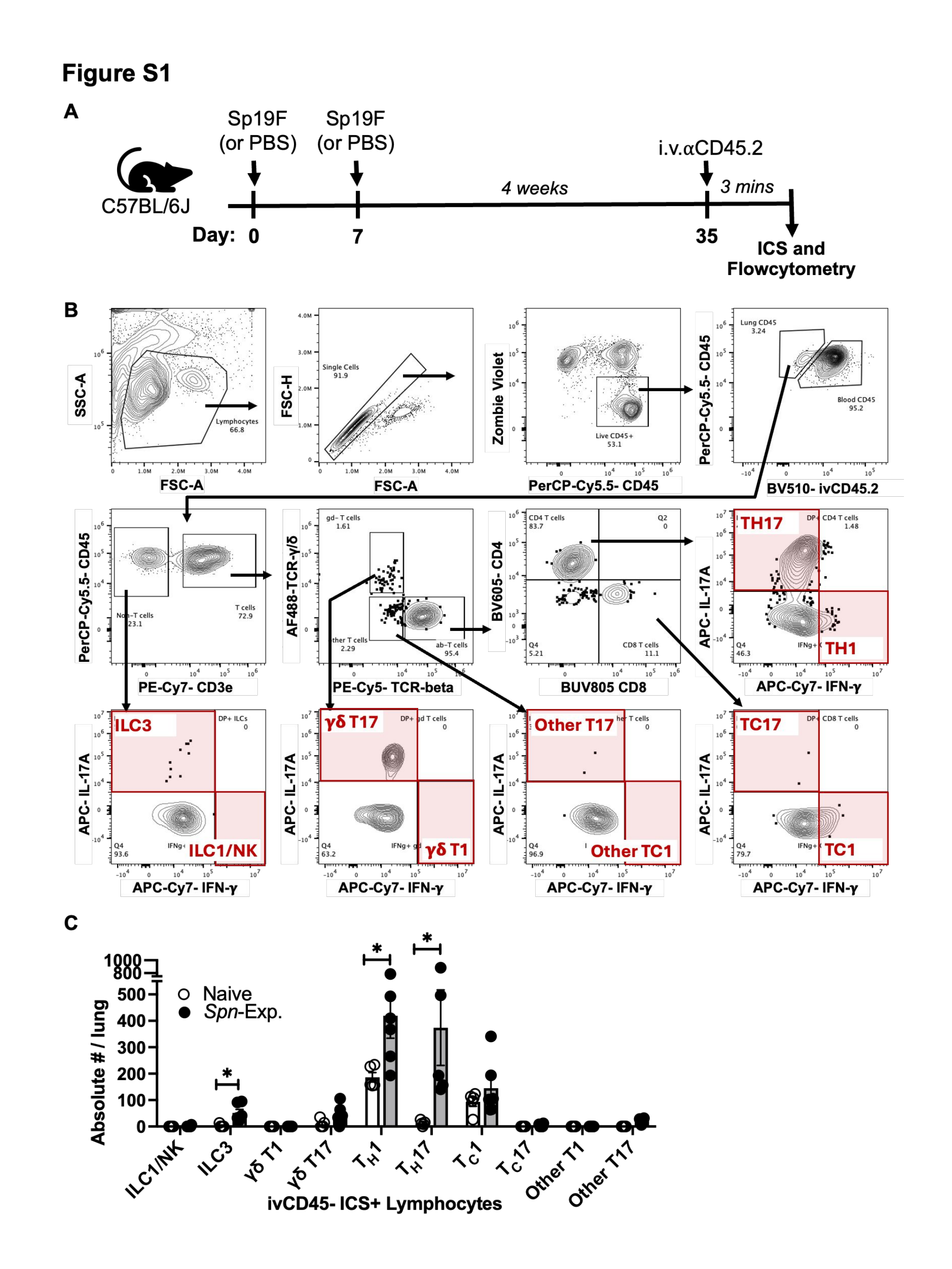

## Slide 2
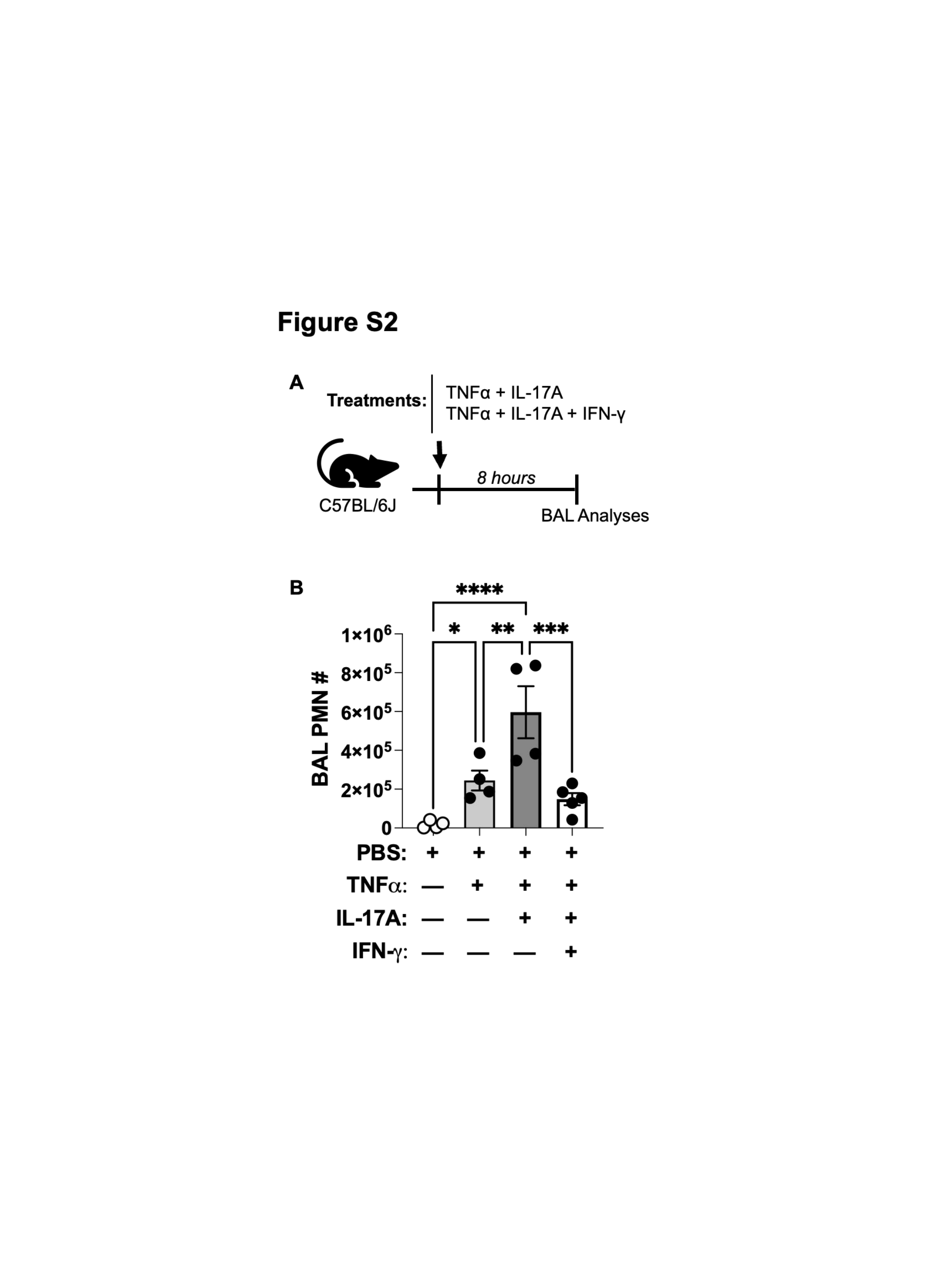

## Slide 3
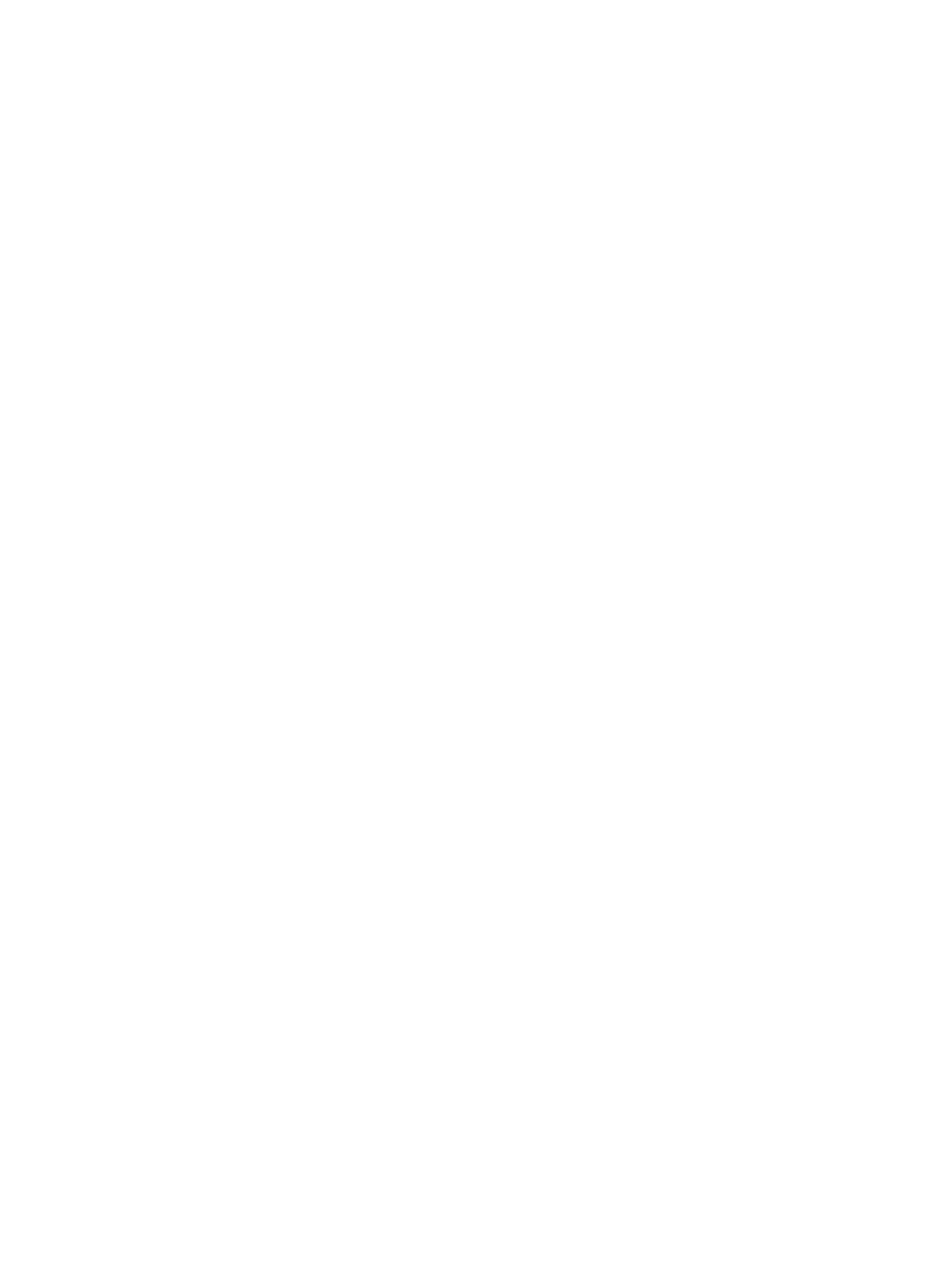

## Slide 4
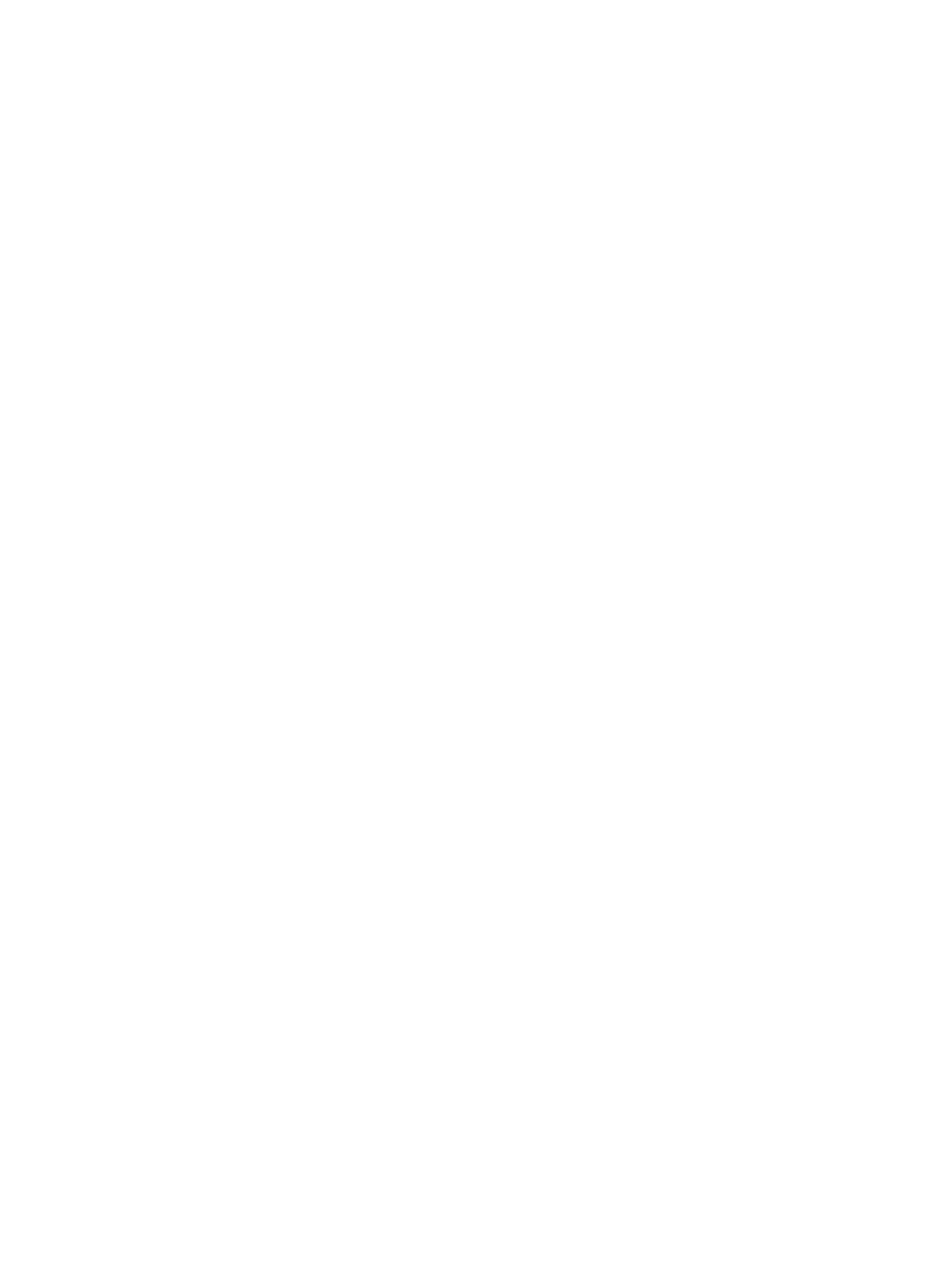

## Slide 5
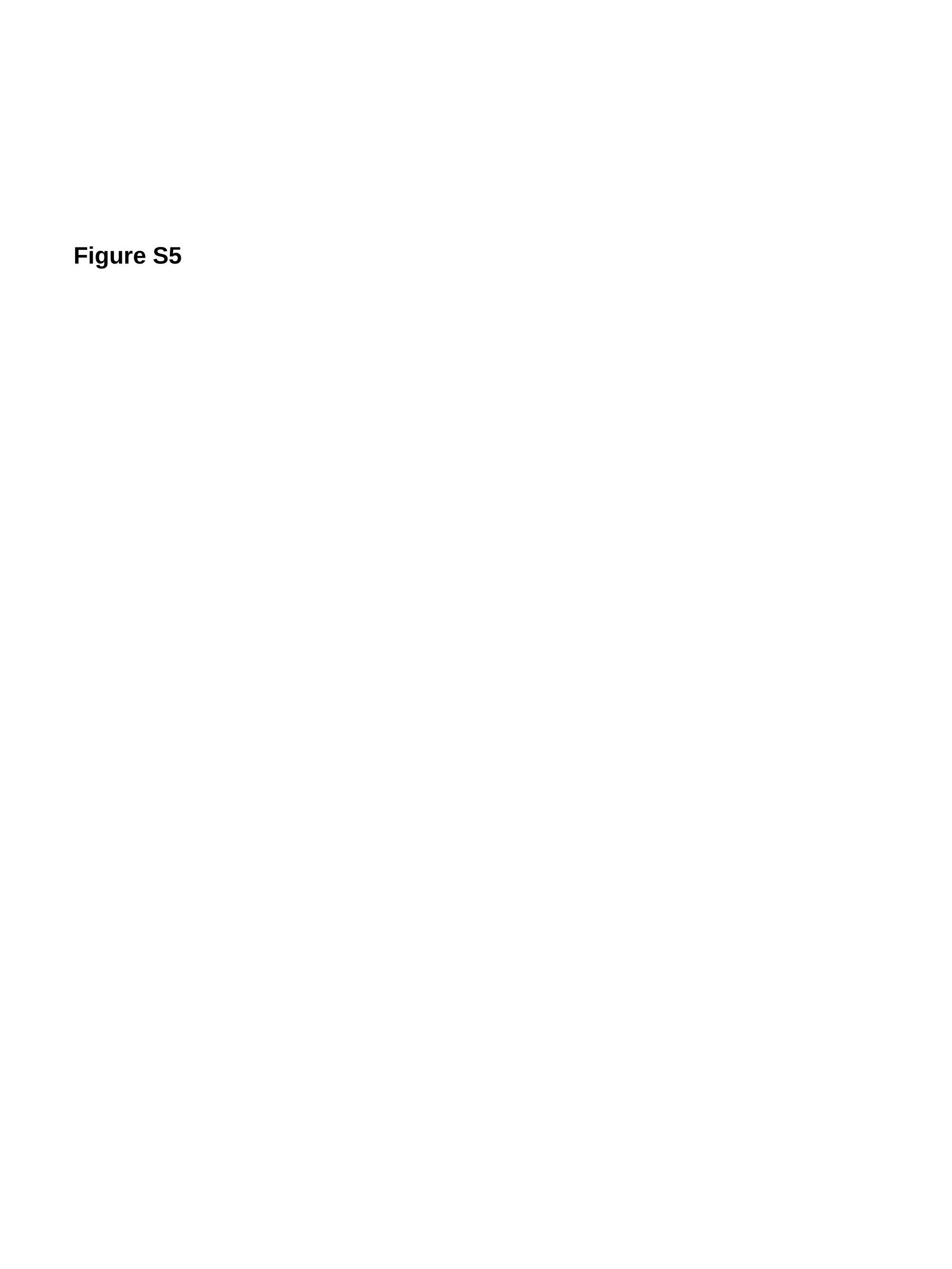

Figure S5

## Slide 6
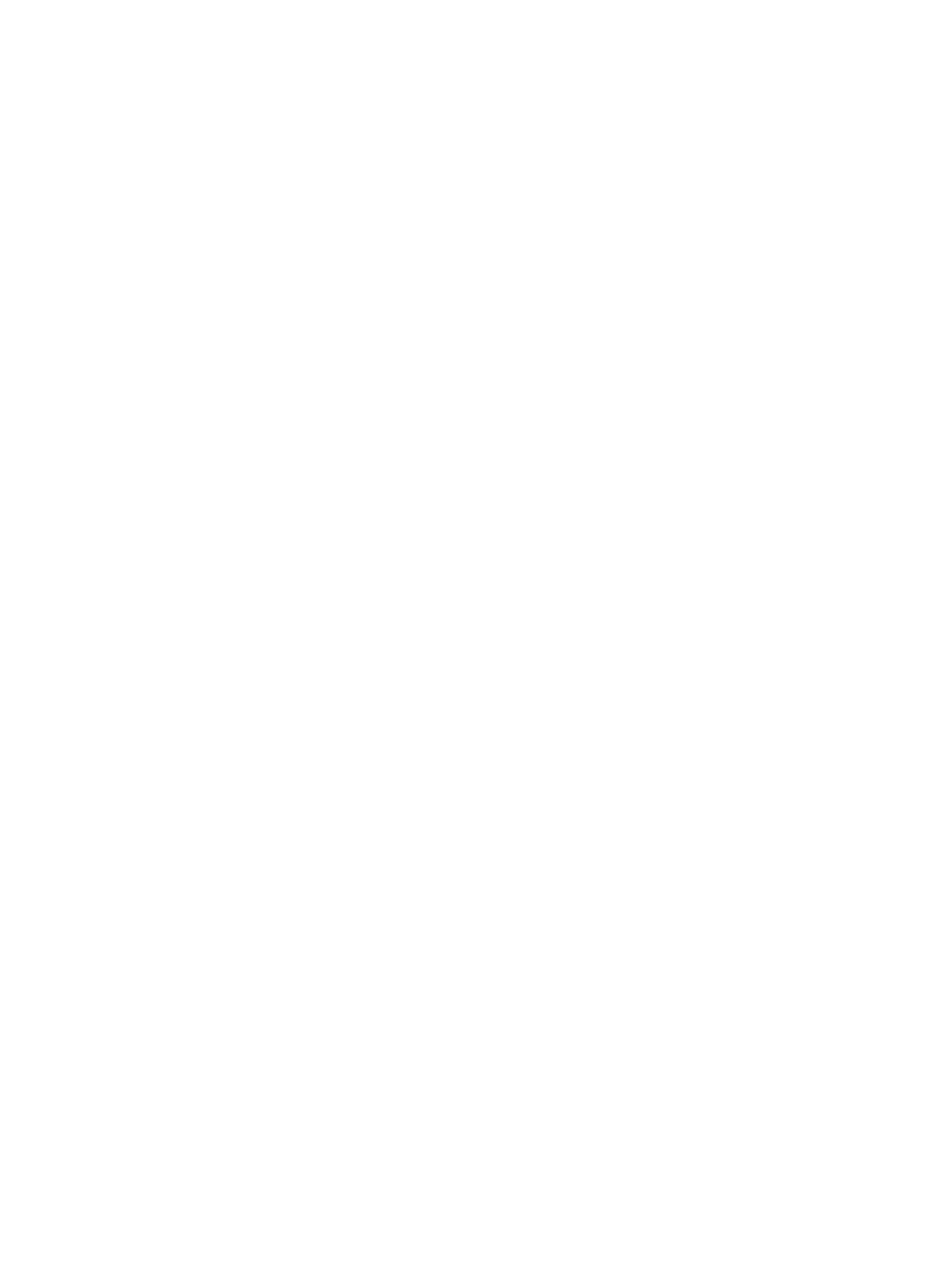

## Slide 7
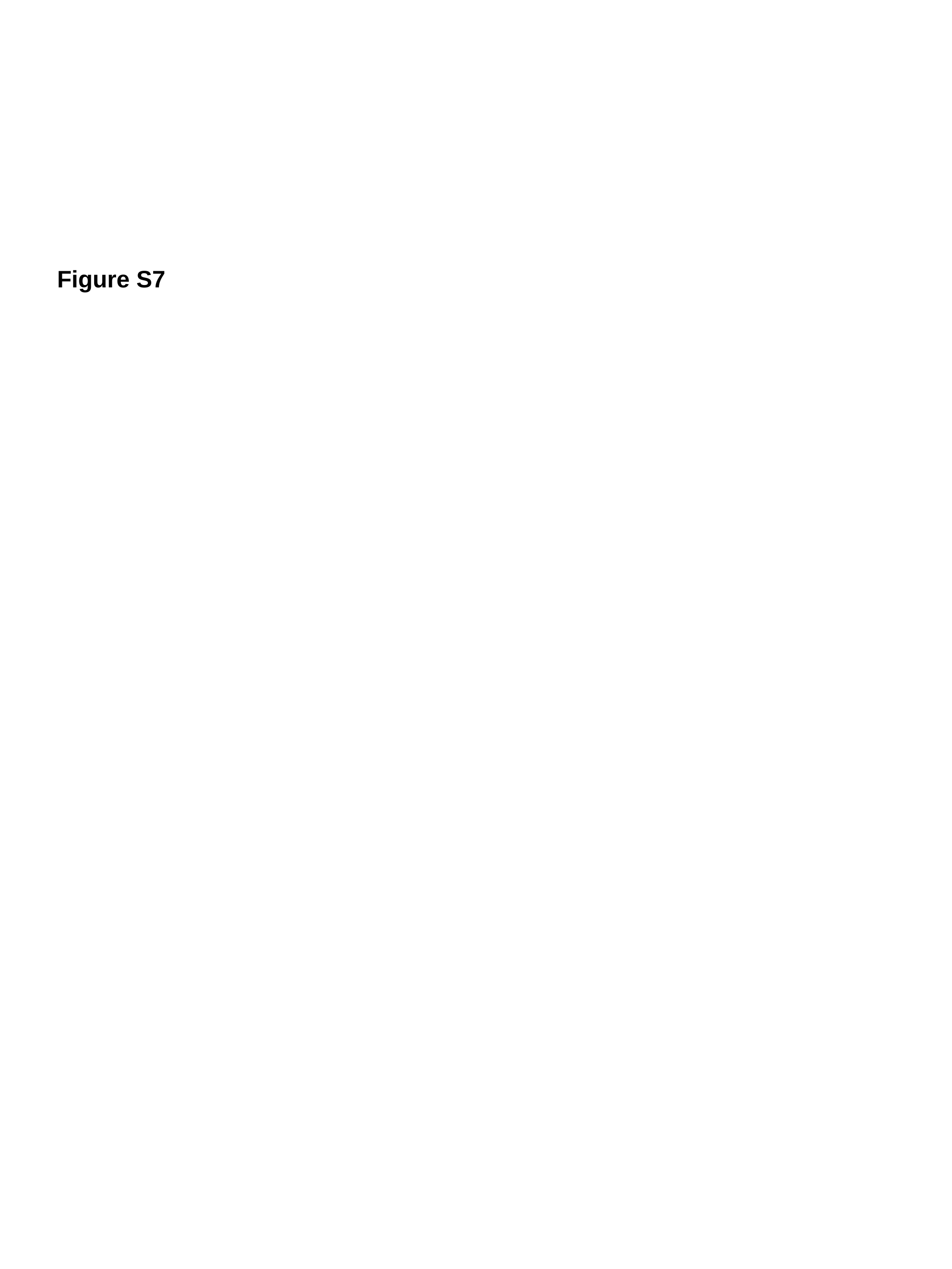

Figure S7

## Slide 8
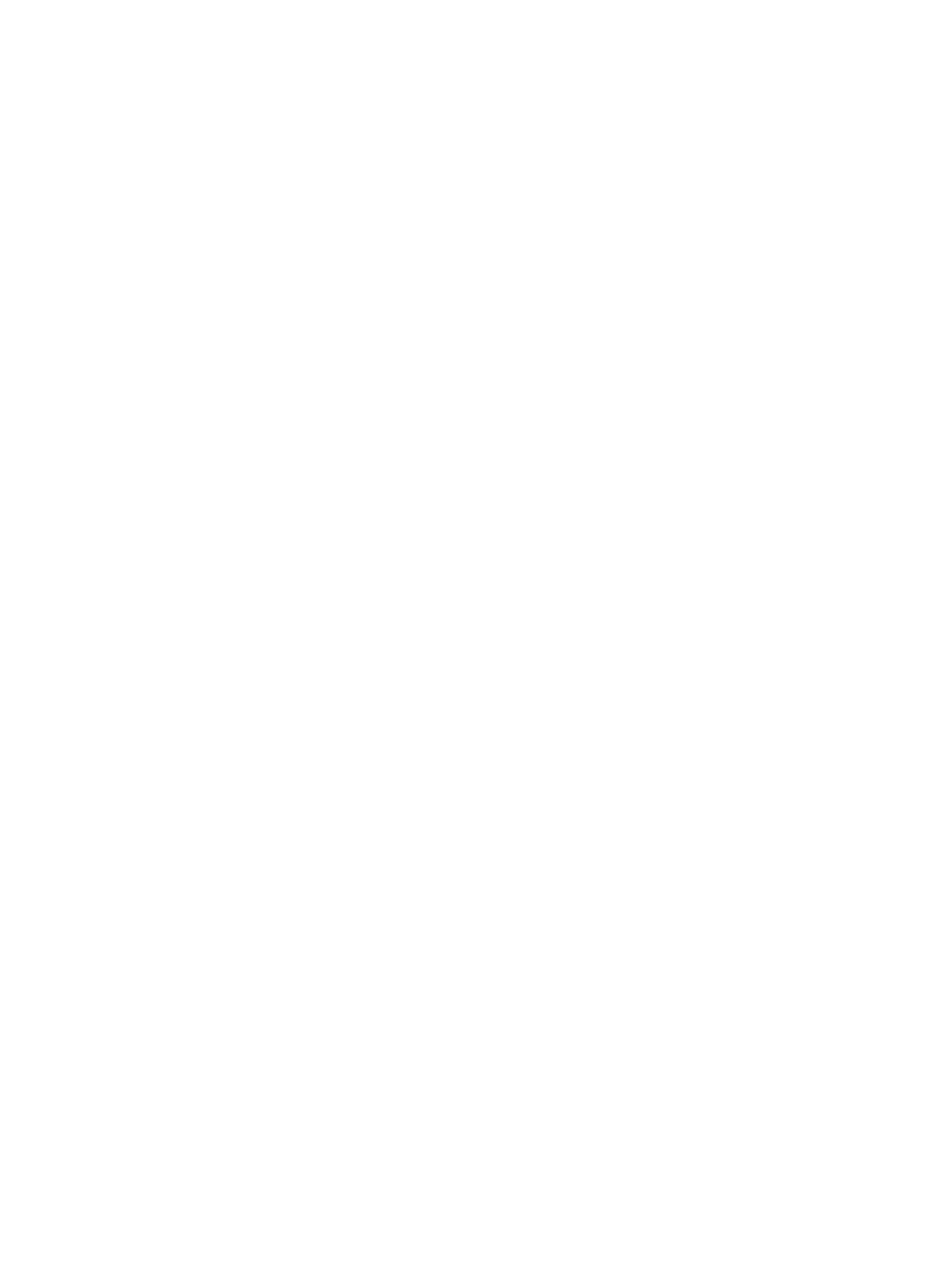

## Slide 9
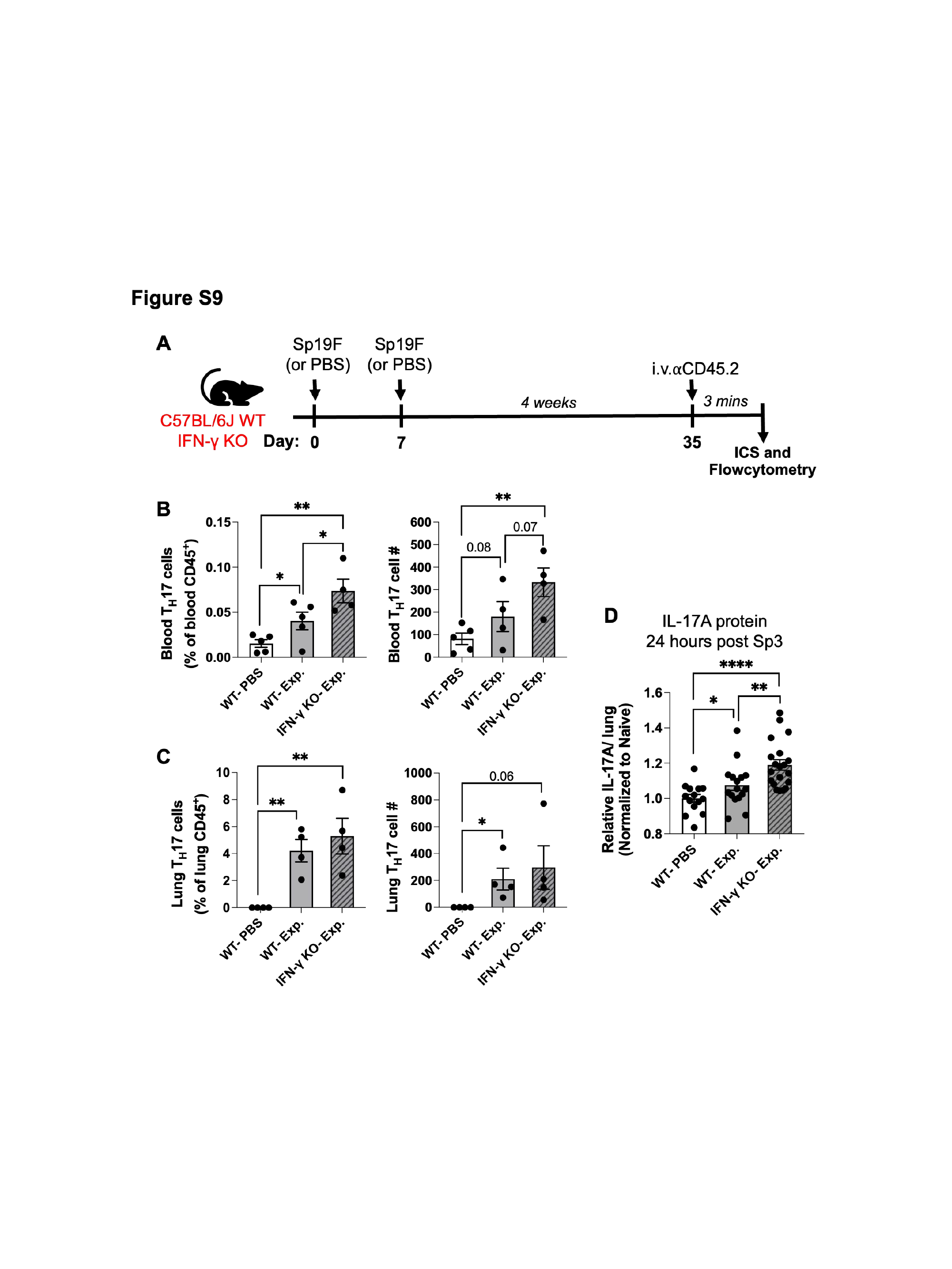

Supplement: Supplementary Figure S1 — Spn-experience seeds murine lungs with tissue-resident TH17 and TH1 TRM cells. (A) Schematic of experimental model used. To accurately discriminate lung resident lymphocytes that are extravascular from circulating lymphoyctes that are intravascular, anesthetized mice administered 2μg anti-CD45.2 antibody 3–5 min prior to lung collection. (B) Flow cytometry gating strategy for distinguishing various cytokine-secreting lung resident lymphocyte populations as identified using intracellular cytokine staining (ICS). (C) Absolute numbers of ICS-positive tissue-resident (ivCD45-) lymphocytes in naïve versus Spn-experienced lungs. Unpaired t test. p value: *≤ 0.05. All data have n≥3 mice, 2 experiment, mean ± SEM. [file SupplementaryFile1.pptx]
